# Local knowledge, practices, challenges of ethnopharmacologically used medicinal plants in Benin and implications for brain illnesses

**DOI:** 10.1038/s41598-023-46647-2

**Published:** 2023-11-13

**Authors:** Godfried Dougnon, Victorien Tamègnon Dougnon, Jean Robert Klotoé, Eric Agbodjento, Dannialou Zoumarou, Boris Lègba, Hornel Koudokpon, Phénix Assogba, Leena Hanski, Eléonore Yayi Ladékan

**Affiliations:** 1 Department of Neuroscience of Disease, Brain Research Institute, Niigata University, Niigata, Japan; 2 Research Unit in Applied Microbiology and Pharmacology of natural substances, Research Laboratory in Applied Biology, Polytechnic School of Abomey-Calavi, University of Abomey-Calavi, Abomey-Calavi, Benin; 3 Faculty of Health Sciences, University of Abomey-Calavi, Cotonou, Benin; 4 Drug Research Program, Division of Pharmaceutical Biosciences, Faculty of Pharmacy, University of Helsinki, Helsinki, Finland; 5 Laboratory of Pharmacognosy and Essential Oils, Institute of Applied Biomedical Sciences, University of Abomey-Calavi, Cotonou, Benin

**Keywords:** Plant sciences, Health care

## Abstract

Traditional medicine (TM) is a significant resource for primary healthcare management all over the world, and principally in Africa. Quality improvement activities that promote evidence-based practices and the integration of traditional medicine into primary healthcare systems can help improve the quality of patient care. In the Republic of Benin (West Africa), traditional medicine practitioners (TMPs) provide different treatments and ways of use, depending on the ailments and the medicinal plants used. The present study aimed at documenting the knowledge, attitudes and practices of Beninese TMPs regarding the use of medicinal plants and the challenges associated with their activities. A focus group survey was conducted using semi-structured interviews with a sample of 91 TMPs in 8 departments of the Republic of Benin. The respondents had an average age of 50 years old and belonged to various categories of TMPs. Medicinal plants are harvested depending on the season and time of the day, and are dried in the shade before being used as decoctions or infusions. Nevertheless, the majority of TMPs do not conduct the necessary tests for quality control, efficacy or toxicity of the proposed remedies, which raises several scientific interrogations, particularly for the treatment of mental and brain-related disorders. Among ~ 110 plants used in the treatment of several pathologies, 66 were revealed as threatened species. The challenges faced by TMPs are mainly material, financial and technical difficulties. The present study reports the importance of intervention to modernize TM practices in Benin. Quality improvement could enhance healthcare delivery and provide support for evidence-based interventions aimed at addressing behavioral, social, and environmental determinants of health.

## Introduction

Traditional Medicine (TM) is the combination of health practices, knowledge, and beliefs that encompass the use of plants, animal parts, minerals, spiritual therapies, techniques, and manual exercises for the purposes of treating, diagnosing, and preventing diseases, as well as maintaining overall health^1^. In developing countries, TM is the primary form of healthcare for over 80% of the population^2,3^, and its significance in primary healthcare has been acknowledged by the Declaration of Alma-Ata (1978) and the guidelines of the Regional Health Policy for All in the twenty-first century^4^. Additionally, the World Health Organization (WHO) advocates for the integration of TM into national health systems and policies to enhance access to care and promote health^1^. In the Republic of Benin (West Africa), ~ 70% of the population relies on TM for their primary healthcare, for several reasons such as the geographical accessibility of herbal remedies, their affordability, and the prevalence of traditional beliefs that promote this practice, especially in the southern part of the country^5–7^. Despite the establishment of laws and organizations to regulate the sector since the late 1990s, TM integration into modern health systems is still in progress^5^.

Further, the lack of scientific evidence regarding the chemical composition and pharmacological properties of Beninese medicinal plant preparations, the limited knowledge, traditional attitudes, and unsound practices of traditional medicine practitioners (TMPs) are numerous obstacles to the integration of traditional medicine into modern health systems^8^. To address these shortcomings, WHO has implemented a TM strategy for the period of 2014–2023, aiming to promote the safe and effective utilization of TM by focusing on regulation, research, and integration into the existing health system^1^. There are many TMPs in the southern part of Benin who provide diverse sorts of treatments and preparations for populations^3^, principally because of their proximity to the local people and the relatively low cost of treatments in comparison to modern medicine^9^. Therefore, the present study aims at documenting the knowledge, attitudes, and practices of TMPs in Benin regarding the use of medicinal plants in Benin. Particularly, it focuses on important points such as the harvesting and processing of medicinal plants, the preparation and conservation of medicinal recipes, and the challenges associated with Beninese TM promotion, with a regard toward the consequences of such practices on mental and brain-related ailments.

## Methods

### Data collection

We used a semi-structured interview method for data collection, as previously described with slight modifications^10^. Briefly, a questionnaire was generated using the Kobocollect application^11^. The questionnaire can be accessed via: https://kf.kobotoolbox.org/#/forms/a8DbT5endnsLu5PfYRzsP9/landing. Voluntary consent was obtained from all respondents prior to data collection. The participants were TMPs recommended by the Ministry of Health of Benin through the National Program of Pharmacopoeia and Traditional Medicine. They were selected according to the following criteria: being legally recognized as a TMP in Benin, having developed at least one herbal product, being proficient in understanding and speaking French and willingly agree to participate in the study.

**Figure 1:**
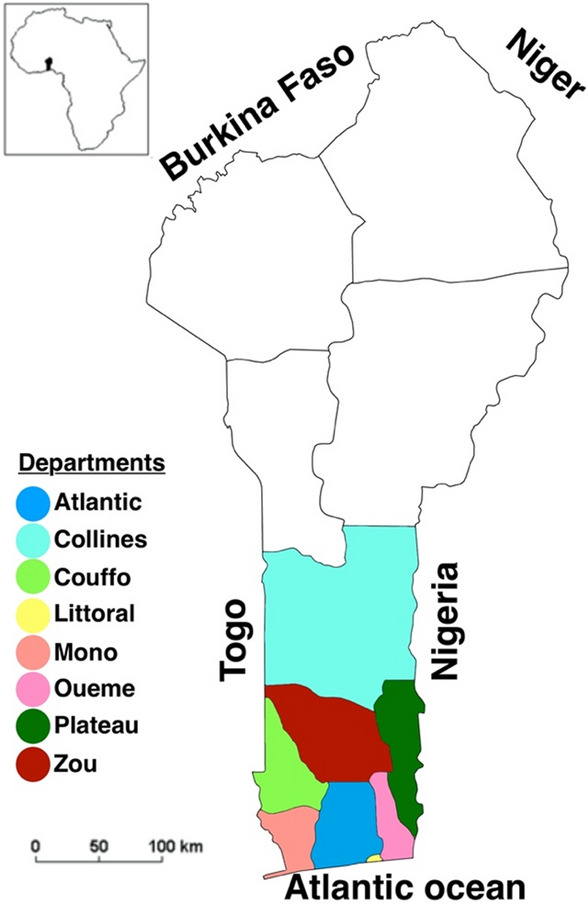
Surveyed regions in the Republic of Benin. The map shows the Republic of Benin with 8 departments surveyed mentioned.

The respondents included members from the two major federations of TMPs in Benin, namely the National Federation of Traditional Medicine Actors in Benin (FANAMETRAB) and the National Federation of Traditional Medicine in Benin (FENAMETRAB). A total of 91 respondents from the departments of Zou, Mono, Couffo, Atlantic, Littoral, Oueme, Collines, and Plateau in Southern Benin (Fig. 1) were included in the study. The data collected from the participants encompassed various aspects, such as socio-demographic information, knowledge, attitudes, and practices related to the harvesting of medicinal plants, techniques for preparing and preserving medicinal recipes, existing regulations regarding the production and distribution of herbal products in Benin, as well as the challenges encountered by TMPs in their professional endeavors.

### Data analysis

The descriptive variables were expressed as numbers and percentages for the entire study population. Statistical analysis was performed using Prism software version 9. Plants local and scientific names were confirmed using the analytical Flora of Benin^12^ and WFO Plant List (https://wfoplantlist.org/plant-list). Additionally, to validate the status of the most threatened species mentioned by TMPs, the IUCN Red List of Threatened Species (https://www.iucnredlist.org/) was utilized. Furthermore, the pathologies commonly treated by TMPs were categorized and classified according to the International Classification of Primary Care–3rd Revision (ICPC-3) of the World Health Organization (https://www.icpc-3.info/).

### Ethics approval and consent to participate

All experimental protocols were approved by the Review Board of Polytechnic School of Abomey-Calavi, University of Abomey-Calavi, Benin. All methods were carried out in accordance with relevant guidelines and regulations. Informed consent was obtained from all TMPs for publication of identifying information/images in an online open-access publication.

## Results

### Socio-demographic characteristics of respondents

**Figure 2:**
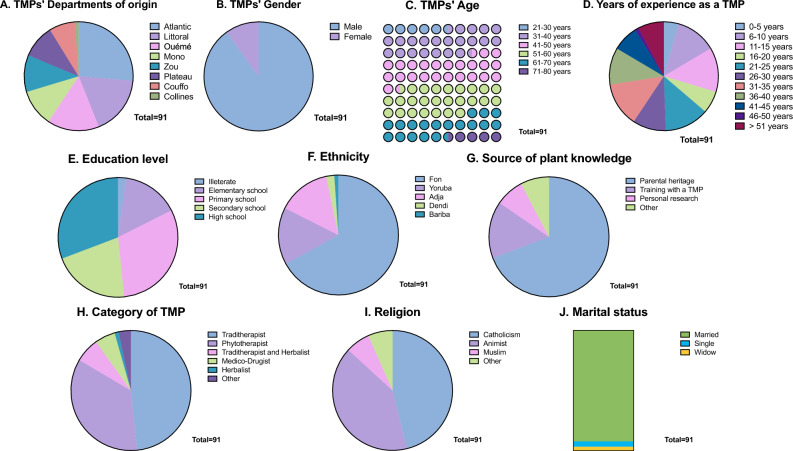
Socio-demographic characteristics of TMPs. (**A**) Department of origin; (**B**) Gender; (**C**) Age; (**D**) Number of years of experience as a TMP; (**E**) Education level; (**F**) Ethnicity; (**G**) Origin of knowledge; (**H**) Category of TMP; (**I**) Religion; (**J**) Marital status.

As presented in Fig. 2, a total of 91 traditional healers from 8 departments in Benin took part in the study. They were mainly male (90%), with an average of 41–60 years (49.5%), and had between 11 and 35 years of experience. Most of the participants were from the Atlantic department (26.4%), with the ethnicity Fon (67%) being majoritarily represented. This indicated that men in the middle age deliver traditional treatments to the population, and most of them were from the principal ethnicity of Benin. Importantly, these results are in accordance with previous reports showing that knowledge is transmitted from generation to generation, and usually to men^3,9,13^.

### Medicinal plants harvesting practices, preparation and conservation

As presented in Table 1, TMPs in Benin primarily collect medicinal plants depending on the season and time of day. The majority of practitioners dry medicinal plants in the shade and use the dried plant parts in their medicinal recipes, which is common practice in several parts of the world^3,14^. However, depending on the purpose of the treatment, drying in the sun is also an alternative method. More, ~ 90% of TMPs harvest and use endangered or threatened plant species, notwithstanding their critical situation. However, to maintain species perpetuation, parts of plants are conserved dried or reduced in powder for future use, which lessens collection of a new plant material.

**Table 1:** Medicinal plants harvesting and processing methods.

	Number of responses	Percentage (%)
Harvesting criteria		
Season of the year	61	33
Time of day	49	27
Quality of the plant (normal physical aspect)	34	19
Maturity of the plant part used	30	16
No particular criteria	9	5
Method of drying		
Under the sun	39	30
In the shade	83	65
Others	6	5
Determinants for drying method		
Disease to be treated	47	35
Plant organ used	49	36
Patient health status	14	10
Random	9	7
Others	17	13
Reasons for drying the plant material		
Reduce water content of the plant	33	25
Prevent fungal growth	32	24
For preservation	66	50
Threatened or endangered species awareness		
Yes	79	87
No	12	13
Threatened or endangered species usage		
Yes	77	85
No	14	15
Plant organs conservation for future use		
Never	3	3
Rarely	16	18
Often	72	79
Form of conservation		
Fresh	16	10
Dried	66	39
Reduced into powder	62	37
Calcinated	24	14

This table describe the criteria for harvesting medicinal plants and their different handling methods.

TMPs reported knowledge of 110 plant species, among which 66 were confirmed as threatened or endangered species upon verification against the IUCN Red List of Threatened Species database (https://www.iucnredlist.org/). These plant species were categorized into 5 levels: data deficient, least concern, endangered, near threatened or vulnerable (Supplemental Table 1).

### Regulation and distribution of herbal health products in Benin

**Figure 3:**
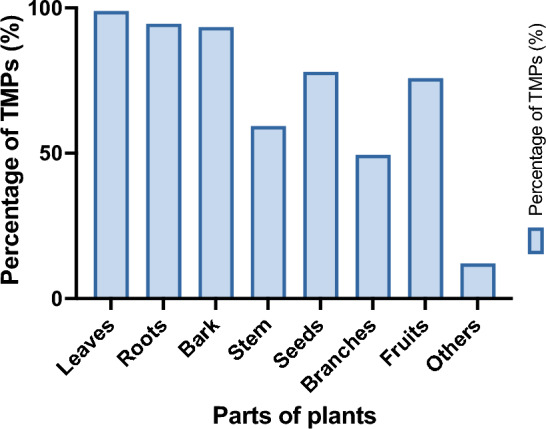
Parts of plants used by TMPs. The graph shows the percentage of TMPs using each part of plants. Leaves, roots and barks were the most used parts of plants.

We next asked about the knowledge of TMPs regarding the processes of preparing and preserving medicinal recipes, evaluating the effectiveness of herbal products, and the regulation and distribution of herbal health products. As presented in Fig. 3, we report that the majority of TMPs use leaves (98.90%), as plant material, followed by roots (94.50%) and bark (93.4%). It is important to mention that many of them deliver their products to the patients without verifying the efficacy or toxicity. Usually, they guaranty treatment efficacy and feedback is obtained from the patient (Table 2).

**Table 2:** Knowledge about the processes of preparing and preserving medicinal recipes and the regulation and distribution of herbal health products.

	Number of TMPs	Percentage (%)
Evaluation of TM efficacy		
Tests in laboratory	23	25
Get feedback from patients	57	63
Do not check	4	4
Others	7	8
Marketing authorization procedures awareness		
Yes	60	66
No	31	34
Procedures for obtaining marketing authorization procedures		
Formulate the drug and put it on the market	6	7
Formulate the drug, do the necessary analysis and put the drug on the market	11	12
Formulate the drug, do the analysis and apply for and obtain a pre-marketing authorization	31	34
Do not know	40	44
Others	3	3

This table shows informations about the procedures for obtaining a marketing authorization for commercial products.

Regarding the regulatory procedures for the production and distribution of herbal products, ~ 44% of the respondents stated that they had no knowledge in this area. Among those who were aware of the regulations, some were not familiar with all the steps involved in obtaining registration for their product, such as formulating the drug, conducting analysis, and obtaining a marketing authorization. Those regulatory practices in Benin are under the supervision of the Benin Agency for Medicines and Other Health Products (Former Beninese Agency of Pharmaceutical Regulation, https://www.abrp.bj/). This agency sets up a national committee which receives the applications for marketing authorization for improved TM offered by TMPs. The committee is based on the directives of Order 017 of February 15th, 2017 relating to the modalities of approval of plant-based medicines in Benin. Indeed, TM products are classified into four categories according to the method of preparation, the indication and the degree of innovation. While category 1 is constituted of any medicine prepared by the TMP for a patient according to given characteristics, category 2 comprises any medicine from the traditional pharmacopoeia and used in the community (improved TM). Category 3 concerns any herbal medicine from research institutes or the pharmaceutical industry. Medicines prepared by approved manufacturing structures or pharmaceutical industries are placed in category 4. Compliance with the conditions for practicing TM and the regulations governing the manufacture of herbal medicines is a sufficient requirement for category 1. Precise conditions are set for the approval of products belonging to categories 2, 3 and 4. Valid for a period of 5 years, and renewable at the request of the TMP, the marketing authorization is granted to any medicinal preparation with clear commercial name, formula, presentation, indications and contraindications. More, the application file for marketing authorization is constituted of the name, form, dosage and presentation of the drug and includes an administrative file, a pharmaceutical file and a toxico-clinical file, according to article 9 of the decree. However, it is important to note that the documents constituting the files are specific to each category of medicines. As for the registration fees to be paid by the applicant, it is required, according to article 14, the payment of a sum of one hundred thousand (100,000 XOF) for category 2 drugs, one hundred and fifty thousand (150,000 XOF) for category 3 medications and two hundred and fifty thousand (250,000 XOF) for those in category 4. Double the amount per category is required for foreigners. The marketing authorization application files are subject to an administrative evaluation, a technical evaluation by the committee of experts. The applications are evaluated according to five modules, notably that relating to administrative and product information, the technical document, the product quality document as well as documents on clinical trials (pharmacological and toxicology documentation, clinical documentation, bioequivalence data or dissolution tests for a medicine under generic name). Obtaining an marketing authorization gives the right to registration of the medicine concerned on the national nomenclature and to its distribution through the formal circuit.

### Medicinal plants usage and diseases treated

Respondents reported treating various diseases and symptoms, which were categorized into 14 systems based on ICPC-3 (https://www.icpc-3.info/), as presented in Table 3. ICPC-3 is a globally recognized concise person-centered health issues classification for Primary Care that provides consistent terminology and coding for diseases, thus making the findings in our study more reliable and comparable with existing works.

**Table 3:** Diseases/symptoms treated by TMPs and their classifications.

Diseases/symptoms indicated by TMPs	System according to ICPC-3
Measles, smallpox, chickenpox, malaria, tuberculosis, leprosy	General
Mycoses, boil or carbuncle	Skin
Gonorrhoea, syphilis, benign prostatic hypertrophy, infertility or subfertility, menopause, menstrual pain, sexual dysfunction	Genital system
Urinary calculus	Urinary system
Premature ejaculation, amnesia, anxiety, depressive disorders, schizophrenia, mood disorders, disorders specifically associated with stress, delirium, dementia	Psychological, mental and neurodevelopmental
Cholera, typhoid, duodenal ulcer, cirrhosis of liver, dental caries, diarrhoea, haemorrhoids, gastric ulcer, nausea, vomiting, gastric pain, constipation	Digestive system
Vertigo or dizziness, paralysis and weakness, epilepsy or seizures, headache, cerebral palsy	Neurological system
Muscle pain	Musculoskeletal system
Cough, acute sinusitis, acute respiratory distress syndrome, asthma, acute nasopharyngitis	Respiratory system
Hypertension, angina pectoris, hypotention, pulmonary embolism	Circulatory system
Diabetes type I or II, overweight, obesity	Endocrine, metabolic and nutritional system
Asymptomactic human immunodeficiency virus-infection, anemia, coagulation defects	Blood, blood-forming organs and immune system
Otitis externa	Ear
Cataract	Eye

According to ICPC-3, the main diseases treated by TMPs in our study and their classification are mentioned.

A total of 110 medicinal plants belonging to 49 botanical families were reported to treat these ailments (Supplemental Table 2). Interestingly, diseases of the neurological system, and psychological, mental and neurodevelopmental disorders were among the main cited diseases. This indicated that TMPs deliver a broad range of herbal products for various ailments, and as further discussed, special attention should be given for diseases affecting the brain, which is arguably the most important organ of the body.

### Attitudes toward methods for harvesting medicinal plants, preparing and preserving medicinal recipes and regulating the production and distribution of herbal products

TMPs demonstrated awareness of the importance of sustainable practices to prevent the extinction of medicinal plant species. They reported engaging in activities such as planting medicinal plants in dedicated gardens and refraining from collecting certain plant organs that could jeopardize the survival of the species. However, the lack of quality control and stability control of their products is a concerning issue, as it could result in the production of ineffective or potentially harmful medicinal products. Additionally, traditional herbal preparations are mostly absent from pharmacies and drugs stores, due to the lack of scientific evidence and regulatory limits which reflects the challenges faced by TMPs in promoting and supporting their herbal products (Table 4).

**Table 4:** Attitudes toward medicinal plant harvesting practices, preparation and preservation of medicinal recipes, and regulation and distribution of herbal health products.

	Number of responses	Percentage (%)
Methods to ensure the perpetuation of medicinal plants		
Grow medicinal plants in garden	62	40
Do not harvest all the plant species available on a harvesting site	37	23.9
Do not harvest critical plant organs such as roots and bark	8	5.2
Conservation in a botanical garden	41	26.5
No particular method	7	4.5
Quality control test of the herbal preparations/products		
Performed	38	41.8
Not performed	53	58.2
Stability control test of the herbal preparations/products		
Performed	30	33
Not performed	61	67
Standardization of the herbal preparations/products formulation		
Performed	50	54.9
Not performed	41	45.1
Commercialization of the herbal preparations/products		
Commercialize	34	37.4
Do not commercialize	57	62.6
Possession of a marketing authorization		
Yes	13	14.3
No	78	85.7
Reasons for not having a marketing authorization		
Do not know how to get it	9	9.9
Cannot afford it	64	70.3
Believe it is unnecessary	4	4.4
No particular reason/others	14	15.4

This table reports general attitudes of TMPs regarding medicinal plants from harvesting to final product presentation.

**Figure 4:**
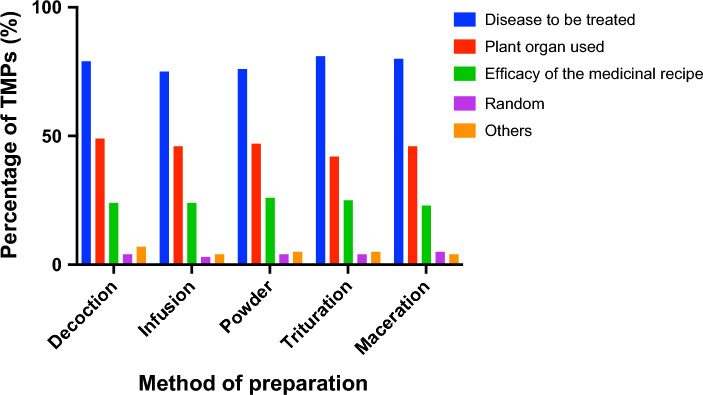
Criteria for choosing different methods of preparation. Each bar represents the percentage of TMPs using a specific method of preparation and the choice criteria.

 Medicinal treatments efficacy depends mainly on the plant used for preparation, but also, on the part of plants used. Chemical compounds are often accumulated in different plant organs^3,15^, which explain the choice of an adequate part of plant and method of preparation. As showed in Fig. 4, ~ 75% of TMPs choose the method of preparation of herbal products based principally on the disease to be treated and ~ 50% relies the part of plant used for the recipe. Only a few respondents (~ 5%) reported selecting a method of preparation randomly.

### Practices related to the harvesting of medicinal plants, preparation and conservation of medicinal recipes.

**Figure 5:**
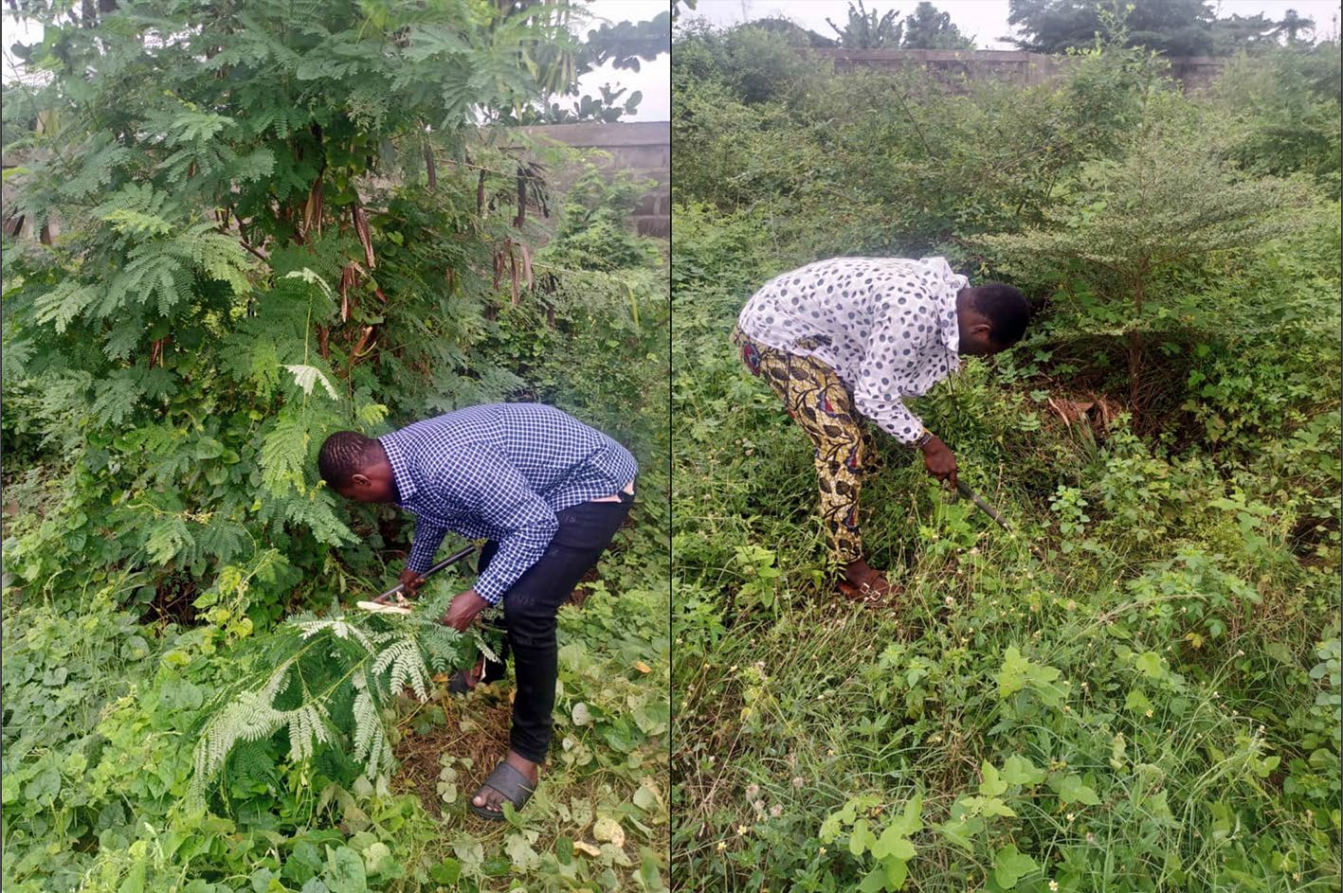
TMPs collecting medicinal plant material at different collection sites. The picture shows 2 TMPs harvesting medicinal plants at different locations.

Harvesting methods employed by TMPs are the collection of specific part of plants such as leaves, barks or roots, or the entire plant uprooting (Table 5). Figure 5 shows TMPs collecting fresh plant material at different collection sites in Cotonou (Littoral department). The most frequently utilized solutions for medicinal preparation are water, alcohol, and honey.

**Table 5:** Practices related to the harvesting of medicinal plants, preparation and conservation of medicinal recipes..

	Number of responses	Percentage (%)
Methods of harvesting plant species		
Harvest the whole plant without roots	27	18.9
Harvest specific plant organs	86	60.1
Uprooting	30	21
Harvesting technique		
No particular precaution	9	9.9
Do not collect all the species at a site	82	90.1
Solvents used for the preparation of the medicinal recipes		
Water	86	27.5
Alcohol	73	23.3
Water-Alcohol mixture	19	6.1
Honey	72	23
Oil	49	15.7
Others	14	4.5
Storage of the medicinal recipes		
Protect from heat	46	28.4
Airtight	39	24.1
With heat	11	6.8
Cold	8	4.9
Using chemical preservatives	6	3.7
Use of alcohol or other beverages	31	19.1
Others	21	13
Products efficacy confirmation		
Laboratory analysis	21	23.1
Patients feedback	44	48.4
Not verified	8	8.8
Personal check	18	19.8
Products toxicity tests		
Laboratory analysis	26	28.6
Patients feedback	17	18.7
Not verified	23	25.3
Personal check	25	27.5
Quality control of the herbal products		
Not conducted	29	31.9
Refer to a laboratory	19	20.9
Personal check of the products	23	25.3
Not tested	20	22
Tests conducted for quality control of herbal products		
Determination of bioactive molecules	9	9.9
Search for microbial contaminants	8	8.8
Search for foreign organic contaminants	8	8.8
Humidity test	7	7.7
Research of metallic trace elements	4	4.4
Search for radioactive contaminants	6	6.6
Testing for pesticides	0	0
Not tested	49	53.8
Stability control tests performed		
pH test	11	12.1
Temperature variation test	8	8.8
Centrifugation test	7	7.7
Dissolution profile test	3	3.3
Color change test	9	9.9
Impurity Level test	4	4.4
Not tested	49	53.8

We report TMPs’ practices of TM from harvesting to distribution of an herbal preparation.

Regarding storage practices, medicinal preparations are primarily kept away from heat and air, with alcohol being used as a preserving agent. The efficacy of medicinal recipes or plant-based products is usually guaranteed by the TMPs who get feedback from the patients. However, laboratory tests for toxicity and quality control tests of products are usually not conducted. Only a few TMPs carry out scientific investigations, to identify bioactive molecules, search for microbial contaminants, and identify foreign organic contaminants.

**Figure 6:**
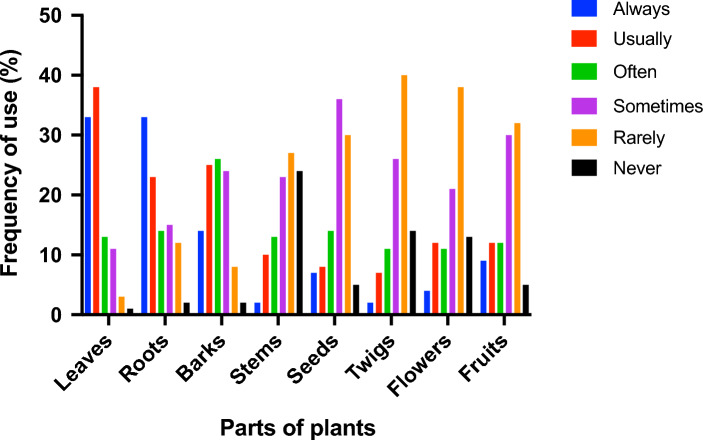
Parts of plants utilization frequency. Frequency of use is reported for the most cited part of plants.

Further, respondents were asked about the frequency of using different plants organs, which revealed that leaves were mainly used, followed by roots and barks (Fig. 6).

### Challenges faced by TMPs and key points to improve the practice of traditional medicine in Benin

TMPs in our study reported principally material, financial and technical difficulties. Accordingly, material challenges included difficulties in supplying raw materials (availability, scarcity of plant species), lack of equipment for conservation, grinding, diagnosis and manufacture of medicinal products and lack of space to serve as a botanical garden for cultivation. Additionally, the collection site for medicinal plants is often razed and used for other purposes by third persons, which threatens species perpetuation. Regarding financial matters, there is a lack of financial support for the purchase of raw materials, and laboratory costs are usually high. Also, there is a need of financial support for the certification of herbal products (analysis and obtaining of products homologation). TMPs also reported a lack of financial means for recruiting qualified people to help them in their activities. Technical difficulties involved the need for training to modernize medicinal plant harvesting practices. More, there is a lack for training to modernize the preparation and conservation practices of medicinal plants. There are also difficulties producing pharmaceutical forms of traditional medicines. Conservation and stability of products are often difficult, and there is a need to improve herbal products designing and strategies for marketing authorization obtention.

While it is important to concede these challenges, adequate proposals and interventions to improve the practice of TM in Benin are required. For example, the government and local authorities could organize periodical training on different approaches of TM practices. The high cost of laboratory analysis prevents some TMPs from conducting necessary tests before delivering to the population, which needs to be addressed. Administrative procedures for marketing authorization obtention should be simplified and allow TMPs to be confident when submitting their demands. Further, the government should facilitate the appropriation of regulatory documents about the production and distribution of plant-based products in the Republic of Benin by implicating more TMPs in decision-making processes. Last, it is crucial to strengthen the collaboration between TMPs, researchers, medical doctors and pharmacists to meet the challenges of the effective promotion of TM.

## Discussion

We reported the practices, knowledge, and attitudes of TMPs in Benin pertaining to the utilization of medicinal plants. The study involved the participation of 91 TMPs, with a majority being male. The average age of the participants was 50 years, and they had 11 to 35 years of experience in traditional Beninese medicine. These findings are in line with data reported in Cameroon^16^, where TM is practiced by older individuals who have accumulated substantial experience over time. According to the respondents, the criteria for harvesting medicinal plants were based on the season and time of day, which conforms with historical practices. This indicates that TMPs in Benin rely on traditional knowledge and ancestral practices when determining the appropriate timing for harvesting medicinal plants. Considering the season and time of day can have an impact on the potency and efficacy of the plants, as certain environmental factors may influence the concentration of bioactive compounds within the plant^3,15^. By adhering to these criteria, TMPs aim to optimize the therapeutic benefits of the harvest medicinal plants. Indeed, WHO^17^ recommends harvesting medicinal plants during optimal seasons or periods to ensure the availability of high-quality material and final herbal products. The timing of harvesting depends on the specific plant part being used, and detailed information regarding the appropriate harvesting time can sometimes be found in national pharmacopoeias, published standards, official monographs, and major reference books. However, in many cases, there is not much scientific evidence behind these recommendations for the metabolite profiles, and TMPs freely decide of the timing of harvesting. It is important to mention that biologically active constituents within medicinal plants can vary depending on the plant's growth and development stage. This variation may include the presence of non-targeted, toxic, or poisonous plant components. Therefore, it is crucial for TMPs to have a comprehensive understanding of the growth stages of medicinal plants and the associated changes in their chemical composition. This ensures the production of safe and effective herbal products while minimizing the potential risks associated with the presence of harmful compounds. Petrovska^18^ proposes that the optimal time for harvesting medicinal plants should be determined based on the quality and quantity of biologically active constituents, rather than solely focusing on the total vegetative yield of the targeted plant parts. Additionally, it is important to consider other factors to ensure optimal harvesting conditions. This includes avoiding harvesting during periods of dew, rain, or exceptionally high humidity. If harvesting must occur in humid conditions, it is crucial to promptly transport the harvested material to a covered area or an indoor drying facility to prevent deterioration caused by increased humidity levels. High humidity can promote microbial fermentation and the growth of molds, which can compromise the quality of the harvested material. To ensure the safety and integrity of the harvested medicinal plant material, it is essential to take precautions and prevent the mixing of foreign materials, weeds, or toxic plants with the intended plant material. Careful attention should be paid to the identification and separation of the targeted medicinal plants to avoid any potential contamination or adverse effects caused by unintended plant species or toxic constituents^17^.

We also report that the raw materials are dried without any prior treatment. Nevertheless, it is recommended that medicinal plant raw materials undergo inspection and sorting before the initial processing stage. This inspection should involve a visual assessment to identify any potential cross-contamination with non-target medicinal plants or plant parts, as well as an evaluation of product quality, non-target plant parts, and the presence of extraneous materials. Organoleptic assessment, including appearance, damage, size, color, odor, and possibly taste, may also be necessary to ensure the overall quality of the raw materials^17^. TMPs indicated that they frequently dry the medicinal plants in the shade, which aligns with standard practice that suggests exposing medicinal plant material to direct sunlight only when necessary for specific drying methods^19–21^. It was observed that most respondents dry medicinal plants with the aim of conserving the species, although a few TMPs apply chemical preservatives, which is not recommended^22^. The use of chemical preservatives should be avoided, and their utilization must adhere to national and/or regional regulations applicable to farmers, collectors, and end-users.

Out of the 110 medicinal plants cited by the TMPs, 66 have been confirmed as threatened according to the IUCN database. This indicated that the TMPs had valid knowledge about medicinal plant species. Among the threatened species, *Tectona grandis* L.f. and *Pterocarpus erinaceus* Poir are recognized as endangered, while *Gossypium herbaceum* L., *Khaya senegalensis* A. Juss., *Vitellaria paradoxa* CF Gaertn, *Garcinia kola* Heckel, and *Prunus africana* (Hook.f.) Kalkman are identified as near threatened or vulnerable. However, it is important to mention that some of these species, such as *Khaya senegalensis* A. Juss., are still being used in medicinal recipes despite their endangered status. It is also worth highlighting that the collection of roots can be particularly damaging to medicinal plants as it can lead to the elimination of the entire species^23^. Leaves can be a better alternative for harvesting certain medicinal plant species, as suggested by Wang et al.^24^. However, we should be aware that the metabolite profile and thus the bioactivity of roots and leaves from the same plant can vary. Harvesting pressures, overexploitation, indiscriminate collection, uncontrolled deforestation, and habitat destruction all contribute to species elimination, but other factors such as range, population size, species diversity, growth rate, and reproductive system also affect elimination risk^25–27^. It is noteworthy mentioning that the harvesting of threatened species for medicinal use can have significant environmental impacts, such as the disappearance of the species and difficulties to preserve the plant for future generations. To promote sustainable harvesting and conservation, it is important to implement measures in order to protect threatened species. Although it is already common practice, TMPs and local populations can also be encouraged to cultivate medicinal plants in their gardens, thus reducing the need to collect wild populations. Undeniably, programs should raise awareness among TMPs and the local population about sustainable practices that can preserve medicinal plant resources for future generations and protect the environment.

The study's findings regarding mental disorders and brain-related disorders within TM practices are of particular interest. All mentioned diseases are of importance, but there is a broad spectrum of neurological conditions, often deeply entangled with cultural and societal factors. TM practices have historically played a key role in addressing neurological and mental health concerns^28^. Therefore, the use of medicinal products to treat these ailments necessitates rigorous scientific evaluation. It is important that the potential efficacy of these natural remedies is assessed through controlled studies, examining their impact on neural pathways, neurotransmitter systems, and cognitive functions. Additionally, the potential for unexpected neurological side effects or interactions with conventional medications should not be underestimated, which warrants necessary pharmacological and toxicological research on traditional remedies. More, investigations on the underlying action mechanisms of these traditional remedies^15,29,30^ should be performed through rigorous studies, and identification of the active compounds implemented to provide valuable insights. This could yield unexpected breakthroughs, such as new compounds with neuroprotective or neuromodulatory properties, thus leading to the development of innovative treatments for neurological and mental health disorders.

Most of the respondents claimed to guaranty efficacy of their products without further testing, which can be risky as some herbal products may demonstrate toxicity in humans^31,32^. Moreover, many respondents do not conduct microbiological and physicochemical quality control or stability testing of their herbal products, which is concerning as the harvesting, preparation and preservation conditions are often not standardized^33,34^. These practices have scientific implications, as they threaten the evidence-based evaluation of traditional treatments. However, solutions can be apported to improve practices. For example, TMPs should receive training in research methodology and testing, delivered in local languages to ensure that all participants truly understand the importance of testing and ensuring quality and safety of their products. Programs to encourage collaboration between scientists and TMPs should be implemented and followed. Further, it is crucial to implement regulations requiring quality and safety testing of natural products at any level of their production.

TMPs also face a number of challenges, including financial difficulties related to obtaining a marketing authorization for their products, as well as a lack of knowledge about the regulations governing the production and distribution of herbal products in Benin. TM in Benin is also often unregulated, and many TMPs have limited or restricted access to modern healthcare resources, not mentioning the lack of formal training. These challenges can lead to concerns regarding the quality and safety of treatments provided. Consequently, patients and healthcare providers may stigmatize TM, making it more challenging for TMPs to provide treatments to the populations. Potential solutions could include regulation and licensing of TMPs, training programs, and encouraging collaboration between traditional and modern medicine, thus ensuring holistic patient care. Governmental support could also be beneficial in reducing the time and costs associated with the administrative procedures. More, support from financial institutions could help lower the costs of laboratory analysis of plant-based products.

Based on these observations, there is an urgent need to promote TM in Benin in a sustainable and evidence-based fashion. We demonstrate that this can be done through the organization of capacity building activities on all topics covered by TM in order to modernize the different approaches and techniques used, strengthening collaboration between TMPs, researchers and modern medicine practitioners in order to improve health outcomes for patients. Also, the government and organizations support would be decisive in the process of marketing authorization obtention, thus making safe and effective herbal remedies available to the population. Nevertheless, while it is important to modernize TM in Benin, cultural significance should also be preserved. This can be achieved by promoting the use of evidence-based approaches to validate traditional remedies, progressively integrating traditional knowledge into modern medicine healthcare, and implementing quality control measures to standardize traditional medicine preparations. Such approaches can help retain the cultural and historical importance of TM and improve its effectiveness and safety. Nevertheless, achieving effective integration of TM in Benin and other African countries may reveal to be challenging due to difficulties in establishing and following policies. However, it is necessary to develop clear regulatory frameworks, and ensure that TMPs and medical doctors are appropriately trained to collaborate. To ease this process, local communities should also be involved in decision-making and gradually integrating TM into modern medicine. As a result, the challenges faced by TMPs in Benin would be addressed, with better access to healthcare and preservation of cultural practices.

## Conclusion

This study aimed to document the knowledge, attitudes, and practices of TMPs in Benin regarding the use of medicinal plants. The findings suggest that Beninese TMPs generally follow recommended practices in terms of collecting medicinal plants and preparing and preserving medicinal recipes. However, some of their attitudes and practices may not ensure product safety and could pose a threat not only to certain plant species, but also to population’s health. More, they face significant challenges in their practice and urgent action is needed to address these issues by providing capacity building activities to enhance the knowledge and skills of TMPs on various themes and modernize the practice of TM in Benin. Support from administrative authorities would be instrumental to alleviate their difficulties in obtaining approval for their traditional preparations.

### Supplementary Information

Supplementary Tables.

## Data Availability

The datasets used and/or analyzed during the current study are available from the corresponding author on reasonable request.
