# Supplementary material for: Local knowledge, practices, challenges of ethnopharmacologically used medicinal plants in Benin and implications for brain illnesses
Source: Sci Rep. 2023 Nov 13;13:19743. doi: 10.1038/s41598-023-46647-2 (PMC10643655; doi:10.1038/s41598-023-46647-2)
Supplement: Supplementary file 1 — Supplementary Tables. [file 41598_2023_46647_MOESM1_ESM.pdf]

Local knowledge, practices, challenges of ethnopharmacologically used medicinal plants in Benin and implications for brain illnesses

Godfried Dougnon[1*], Victorien Tamègnon Dougnon[2*], Jean Robert Klotoé[2], Eric Agbodjento[2], Dannialou Zoumarou[3], Boris Lègba[2], Hornel Koudokpon[2], Phénix Assogba[2], Leena Hanski[4], Eléonore Yayi Ladékan[5]

[1]Department of Neuroscience of Disease, Brain Research Institute, Niigata University, Japan

[2]Research Unit in Applied Microbiology and Pharmacology of Natural Substances, Research Laboratory in Applied Biology, Polytechnic School of Abomey-Calavi, University of Abomey-Calavi, Benin

[3]Faculty of Health Sciences, University of Abomey-Calavi, Benin

[4]Drug Research Program, Division of Pharmaceutical Biosciences, Faculty of Pharmacy, University of Helsinki, Finland

[5]Laboratory of Pharmacognosy and Essential Oils, Institute of Applied Biomedical Sciences, University of Abomey-Calavi, Benin

[*]Correspondence: dougnong@bri.niigata-u.ac.jp; victorien.dougnon@epac.uac.bj.

Supplemental information

Supplemental Table 1. Classification of threatened plant species.

Number	Vernacular names	Scientific names	Family	Red List category
**1**	Tecki	*Tectona grandis* L.f.	Lamiaceae	Endangered
**2**	Kosso	*Pterocarpus erinaceus* Poir.	Fabaceae	Endangered
**3**	Amanga	*Mangifera indica* L.	Anacardiaceae	Data deficient
**4**	Aknari	*Opuntia ficus-indica* (L.) *Mill.*	Cactaceae	Data deficient
**5**	Gbakaya	*Manihot esculenta* Crantz	Euphorbiaceae	Data deficient
**6**	Dotè	*Zingiber officinale* Roscoe	Zingiberaceae	Data deficient
**7**	kpintin	*Carica papaya L.*	Caricaceae	Data deficient
**8**	Ahouatin	*Parkia biglobosa* (Jacq.) R.Br. ex G.Don	Fabaceae	Least concern
**9**	Adité	*Ekebergia capensis* Sparrm.	Meliaceae	Least concern
**10**	Amassou	*Senna alata* (L.) Roxb.	Fabaceae	Least concern
**11**	Hêhêman	*Heterotis rotundifolia* (Sm.) Jacq.-Fél.	Melastomataceae	Least concern
**12**	Kinineman	*Azadirachta indica* A. Juss.	Meliaceae	Least concern
**13**	Gbaguidikpotinwéwé	*Jatropha curcas* L.	Euphorbiaceae	Least concern
**14**	Hêtin	*Zanthoxylum zanthoxyloides* (Lam.) B.Zepernick & Timler	Rutaceae	Least concern
**15**	Acacia	*Senna occidentalis* (L.) Lien	Fabaceae	Least concern
**16**	Détin	*Elaeis guineensis Jacq.*	Arecaceae	Least concern
**17**	Gnanblikpo	*Kigelia africana* (Lam.) Benth.	Bignoniaceae	Least concern
**18**	kpachima	*Moringa oleifera* Lam.	Moringaceae	Least concern
**19**	Orgueilli	*Caesalpinia pulcherrima* (L.) Sw.	Fabaceae	Least concern
**20**	Ayalahado	*Uvaria chamae* P.Beauv.	Annonaceae	Least concern
**21**	Kpédjélékoun	*Xylopia aethiopica* A.Rich.	Annonaceae	Least concern

**22**	Acassia	*Senna siamea* (Lam.) H.S.Irwin & Barneby	Fabaceae	Least concern
**23**	Acajou	*Anacardium occidentale* L.	Anacardiaceae	Least concern
**24**	Adjahè	*Stereospermum kunthianum* Cham.	Bignoniaceae	Least concern
**25**	Aflatovi	*Portulaca oleracea* L.	Portulacaceae	Least concern
**26**	Aboeboe	*Rhipsalis baccifera* subsp. baccifera	Cactaceae	Least concern
**27**	Agbossou ningla	*Voacanga africana* Stapf ex Scott Elliot	Apocynacea	Least concern
**28**	Ahanzo	*Carissa spinarum* L.	Apocynaceae	Least concern
**29**	Aïdan	*Tetrapleura tetraptera* (Schumach. & Thonn.) Taub.	Fabaceae	Least concern
**30**	Akouèman	*Lannea acida* A.Rich.	Anacardiaceae	Least concern
**31**	Assoisoinman	*Dialium guineense* Willd.	Fabaceae	Least concern
**32**	Atindordokpé	*Pseudocedrela kotschyi* Harms	Meliaceae	Least concern
**33**	Aviman	*Cola acuminata* (P.Beauv.) Schott & Endl.	Malvaceae	Least concern
**34**	Ayignan	*Prosopis africana* (Guill. & Perr.) Taub.	Fabaceae	Least concern
**35**	Azouzoui	*Lannea barteri* Engl.	Anacardiaceae	Least concern
**36**	Biwêrê	*Tephrosia vogelii* Hook.f.	Fabaceae	Least concern
**37**	Chorosol assou	*Annona muricata* L.	Annonaceae	Least concern
**38**	Kinkéliba	*Combretum micranthum* G.Don	Combretaceae	Least concern
**39**	Dessleguè	*Newbouldia laevis* (P.Beauv.) Seem.	Bignoniaceae	Least concern
**40**	Goutin	*Erythrina senegalensis* DC.	Fabaceae	Least concern
**41**	Ewédodo	*Rauvolfia vomitoria* Wennberg	Apocynaceae	Least concern
**42**	Fiorman	*Cyperus esculentus* L.	Cyperaceae	Least concern
**43**	Amonman	*Ceiba pentandra* (L.) Gaertn.	Malvaceae	Least concern
**44**	Kinkoutin	*Psidium guajava* L.	Myrtaceae	Least concern
**45**	Honsoukokoe	*Bridelia ferruginea* Benth.	Phyllanthaceae	Least concern
**46**	Wèkèman	*Jatropha multifida* L.	Euphorbiaceae	Least concern
**47**	Kakè	*Burkea africana* Hook.	Fabaceae	Least concern
**48**	Atabouman	*Chamaecrista mimosoides* (L.) Greene	Fabaceae	Least concern

**49**	Gna kpé kpé	*Kigelia africana* (Lam.) Benth.	Bignoniaceae	Least concern
**50**	Kpakpaki	*Rhaphiostylis beninensis* (Hook.f. ex Planch.) Planch. ex Benth.	Metteniusaceae	Least concern
**51**	Kpatador	*Securidaca longepedunculata* Fresen.	Polygalaceae	Least concern
**52**	Kpédjélé	*Lannea acida* A.Rich.	Anacardiaceae	Least concern
**53**	Lissètin	*Blighia sapida* K.D.Koenig	Sapindaceae	Least concern
**54**	Tamarin	*Tamarindus indica* L.	Fabaceae	Least concern
**55**	Hontonzouzin	*Crateva adansonii subsp. odora* (Buch.-Ham.) Jacobs	Capparaceae	Least concern
**56**	Adjikouin	*Guilandina bonduc* var. majus DC.	Caesalpiniaceae	Least concern
**57**	Ahwanglon	*Tribulus terrestris* L.	Zygophyllaceae	Least concern
**58**	Tchivi	*Trichilia emetica* subsp. suberosa J.J.de Wilde	Meliaceae	Least concern
**59**	Vikplonba	*Rourea coccinea* (Schumach. & Thonn.) Hook.f.	Connaraceae	Least concern
**60**	Lokotin	*Milicia excelsa* (Welw.) CCBerg	Moraceae	Near threatened
**61**	Asrosikan	*Irvingia gabonensis* (Aubry-Lecomte ex O'Rorke) Baill.	Irvingiaceae	Near threatened
**62**	Avokanfoun tchèkè	*Gossypium herbaceum* L.	Malvaceae	Near threatened
**63**	Zounza	*Khaya senegalensis* A.Juss.	Meliaceae	Vulnerable
**64**	Limoutin	*Vitellaria paradoxa* CF Gaertn.	Sapotaceae	Vulnerable
**65**	Ahowé	*Garcinia kola* Heckel	Clusiaceae	Vulnerable
**66**	Preunier noir	*Prunus africana* (Hook.f.) Kalkman	Rosaceae	Vulnerable

Supplemental Table 2. List of plants used in traditional treatment of various diseases.

Number	Vernacular names	Scientific names	Family	Number of citations
**1**	Kpatinman	*Moringa oleifera* Lam.	Moringaceae	19
**2**	Tchayo	*Ocimum gratissimum* L.	Lamiaceae	15
**3**	Dessleguè	*Newbouldia laevis* (P.Beauv.) Seem.	Bignoniaceae	14
**4**	Acacia	*Senna siamea* (Lam.) H.S.Irwin & Barneby	Fabaceae	12
**5**	Zounza	*Khaya senegalensis* A.Juss.	Meliaceae	11
**6**	Yinssikin	*Momordica charantia* L.	Cucurbitaceae	11
**7**	Kinkeliba	*Senna occidentalis* (L.) Link	Fabaceae	11
**8**	Klé	*Citrus aurantiifolia* (Christm.) Swingle	Rutaceae	9
**9**	Timan	*Cymbopogon citratus* Stapf	Poaceae	8
**10**	Adjélélé	*Croton gratissimus* var. gratissimus	Euphorbiaceae	7
**11**	Amanga man	*Mangifera indica* L.	Anacardiaceae	7
**12**	Honton zounzoun	*Crateva adansonii* DC.	Capparaceae	6
**13**	Alorvi atoon	*Jatropha multifida* L.	Euphorbiaceae	6
**14**	Hè	*Zanthoxylum zanthoxyloides* (Lam.) B.Zepernick & Timler	Rutaceae	6
**15**	Dotè	*Zingiber officinale* var. officinale Roscoe	Zingiberaceae	6
**16**	Golo	*Cola acuminata* (P.Beauv.) Schott & Endl.	Malvaceae	5
**17**	Koucoman	*Spondias mombin* L.	Anacardiaceae	5
**18**	Vivikannon	*Abrus precatorius* L.	Fabaceae	4
**19**	Kininoutin	*Azadirachta indica* A.Juss.	Meliaceae	4
**20**	Doubaman	*Carica papaya* L.	Caricaceae	4
**21**	Amanvivè	*Gymnanthemum amygdalinum* (Delile) Sch.Bip.	Asteraceae	4
**22**	Gbaguidi kpotinman	*Jatropha curcas* L.	Euphorbiaceae	4
**23**	Dodemakpohwoe	*Rauvolfia vomitoria* Wennberg	Apocynaceae	4
**24**	Kpassaman	*Adansonia digitata* L.	Malvaceae	3
**25**	Wédémégbé	*Ageratum conyzoides* L.	Asteraceae	3
**26**	Adjikouin	*Guilandina bonduc* L.	Fabaceae	3
**27**	Hlinwé	*Phyllanthus amarus* Schumach. & Thonn.	Phyllanthaceae	3
**28**	Atinkin gbadota	*Syzygium aromaticum* (L.) Merr. & L.M.Perry	Myrtaceae	3
**29**	Laurel	*Syzygium racemosum* DC.	Myrtaceae	3
**30**	Ayalayado	*Uvaria chamae* P.Beauv.	Annonaceae	3
**31**	Kpedjelikoun	*Xylopia aethiopica* A.Rich.	Annonaceae	3

**32**	Ahangloo	*Acanthospermum hispidum* DC.	Asteraceae	2
**33**	Gbaguinnan	*Acridocarpus smeathmanni* Guill. & Perr.	Malpighiaceae	2
**34**	Klan madou	*Alchornea cordifolia* (Schumach.) Müll.Arg.	Euphorbiaceae	2
**35**	Chap chap	*Annona muricata* L.	Annonaceae	2
**36**	Artemesia	*Artemisia vulgaris* L.	Asteraceae	2
**37**	Honsoukoué koué,	*Bridelia ferruginea* Benth.	Phyllanthaceae	2
**38**	Orgeuillit	*Caesalpinia pulcherrima* (L.) Sw.	Fabaceae	2
**39**	Amonman	*Calotropis procera* (Aiton) Dryand.	Apocynaceae	2
**40**	Ntchiman	*Citrus aurantium* L.	Rutaceae	2
**41**	Aziman	*Crotalaria retusa* L.	Fabaceae	2
**42**	Curcuma	*Curcuma longa* L.	Zingiberaceae	2
**43**	Ahehe	*Dicoma tomentosa* Klatt	Asteraceae	2
**44**	Kpormié	*Harpagophytum procumbens* DC. ex Meisn.	Pedaliaceae	2
**45**	Heheman	*Heterotis rotundifolia* (Sm.) Jacq.-Fél.	Melastomataceae	2
**46**	Fànnyivi	*Lantana camara* L.	Verbenaceae	2
**47**	Yantotoe	*Launaea taraxacifolia* (Willd.) Amin ex C.Jeffrey	Asteraceae	2
**48**	Asrosikan	*Momordica balsamina* L.	Cucurbitaceae	2
**49**	Kessoukessou	*Ocimum americanum* L.	Lamiaceae	2
**50**	Ahouatin (Néré)	*Parkia biglobosa* (Jacq.) R.Br. ex G.Don	Fabaceae	2
**51**	Avocatin	*Persea americana* Mill.	Lauraceae	2
**52**	Kinkounman	*Psidium guajava* L.	Myrtaceae	2
**53**	Toflougba	*Pteridium aquilinum* (L.) Kuhn	Dennstaedtiaceae	2
**54**	Kpakpakin	*Rhaphiostylis beninensis* (Hook.f. ex Planch.) Planch. ex Benth.	Metteniusaceae	2
**55**	Amansou	*Senna alata* (L.) Roxb.	Fabaceae	2
**56**	Adjahè	*Stereospermum kunthianum* Cham.	Bignoniaceae	2
**57**	Tamarin	*Tamarindus indica* L.	Fabaceae	2
**58**	Wélékpékpé	*Acmella caulirhiza* Delile	Asteraceae	1
**59**	Kivi	*Actinidia chinensis* Planch.	Actinidiaceae	1
**60**	Tonti	*Adenia lobata* Engl.	Passifloraceae	1
**61**	Akouêman	*Aerva lanata* (L.) Juss.	Amaranthaceae	1
**62**	Atakoun	*Aframomum melegueta* K.Schum.	Zingiberaceae	1
**63**	Abiwèlè	*Afrohybanthus enneaspermus* (L.) Flicker	Violaceae	1
**64**	Sabla	*Allium cepa* L.	Amaryllidaceae	1
**65**	Ayo	*Allium sativum* L.	Amaryllidaceae	1

**66**	Aerovera	*Aloe buettneri* A.Berger	Asphodelaceae	1
**67**	Fotêtê	*Amaranthus cruentus* L.	Amaranthaceae	1
**68**	Acajou	*Anacardium occidentale* L.	Anacardiaceae	1
**69**	Ananan	*Ananas comosus* (L.) Merr.	Bromeliaceae	1
**70**	Ahanzo	*Baissea baillonii* Hua	Apocynaceae	1
**71**	Agbégbé	*Cassytha filiformis* L.	Lauraceae	1
**72**	Flawe	*Catharanthus roseus* (L.) G.Don	Apocynaceae	1
**73**	Akaya	*Cleome gynandra* L.	Cleomaceae	1
**74**	Agonkètin	*Cocos nucifera* L.	Arecaceae	1
**75**	Eucalyptus	*Corymbia torelliana* (F.Muell.) K.D.Hill & L.A.S.Johnson	Myrtaceae	1
**76**	Afiô	*Cyperus esculentus* L.	Cyperaceae	1
**77**	Gbadawèn	*Dichrostachys cinerea* subsp. africana Brenan & Brummitt	Fabaceae	1
**78**	Wiinbou	*Diospyros mespiliformis* Hochst. ex A.DC.	Ebenaceae	1
**79**	Détin	*Elaeis guineensis* Jacq.	Arecaceae	1
**80**	Goutin	*Erythrina senegalensis* DC.	Fabaceae	1
**81**	Nonsiwé	Euphorbia hirta	Euphorbiaceae	1
**82**	Tchèkè tchèkè man	*Flueggea virosa* (Roxb. ex Willd.) Royle	Phyllanthaceae	1
**83**	Ahowé	*Garcinia kola* Heckel	Clusiaceae	1
**84**	Koklossou dinkpadja	*Heliotropium indicum* L.	Boraginaceae	1
**85**	Ebé	*Imperata cylindrica* (L.) P.Beauv.	Poaceae	1
**86**	Afaman	*Kalanchoe pinnata* (Lam.) Pers.	Crassulaceae	1
**87**	Gnanblikpo	*Kigelia africana* (Lam.) Benth.	Bignoniaceae	1
**88**	Wugo asu	*Lophira lanceolata* Tiegh. ex Keay	Ochnaceae	1
**89**	Eroun	*Luffa cylindrica* M.Roem.	Cucurbitaceae	1
**90**	Zintintin	*Lycopodiella cernua* (L.) Pic.Serm.	Lycopodiaceae	1
**91**	Atôman	*Malus sylvestris* (L.) Mill.	Rosaceae	1
**92**	Finyin	*Manihot esculenta* Crantz	Euphorbiaceae	1
**93**	Kpofin	*Mezoneuron benthamianum* Baill.	Fabaceae	1
**94**	Loko	*Milicia excelsa* (Welw.) C.C.Berg	Moraceae	1
**95**	Sasalikou	*Monodora myristica* Dunal	Annonaceae	1
**96**	Noni	*Morinda citrifolia* L.	Rubiaceae	1
**97**	Houinsido	*Morinda lucida* Benth.	Rubiaceae	1
**98**	Bananier	*Musa paradisiaca* L.	Musaceae	1
**99**	Akohouman	*Ocimum basilicum* L.	Lamiaceae	1
**100**	Gouton	*Phoenix canariensis* H.Wildpret	Arecaceae	1

**101**	Ayokpè	*Picralima niti*da T.Durand & H.Durand	Apocynaceae	1
**102**	Ayignan	*Prosopis africana* (Guill. & Perr.) Taub.	Fabaceae	1
**103**	Trèdagbokor	*Pupalia lappacea* (L.) Juss.	Amaranthaceae	1
**104**	Kpanwoun	*Senna tora* (L.) Roxb.	Fabaceae	1
**105**	Tinmatin	*Solanum lycopersicum* L.	Solanaceae	1
**106**	Biwêrê	*Tephrosia vogelii* Hook.f.	Fabaceae	1
**107**	Ahwanglon assou	*Tribulus terrestris* L.	Zygophyllaceae	1
**108**	Tégbéssou man	*Vincetoxicum cameroonicum* (N.E.Br.) Meve & Liede	Apocynaceae	1
**109**	Karité	*Vitellaria paradoxa* subsp. paradoxa	Sapotaceae	1
**110**	Lèman	*Ximenia americana* L.	Olacaceae	1
